# Dosing of Antimycotic Treatment in Sepsis–Induced Liver Dysfunction by Functional Liver Testing with LiMAx®

**DOI:** 10.1155/2019/5362514

**Published:** 2019-12-26

**Authors:** Carmen Kirchner, Jasmin Sibai, Elke Schwier, Dietrich Henzler, Claas Eickmeyer, Günther Winde, Thomas Köhler

**Affiliations:** ^1^Department of General and Visceral Surgery, Thoracic Surgery and Proctology, Ruhr University Bochum, Klinikum Herford, Herford, Germany; ^2^Department of Anesthesiology, Surgical Intensive Care, Emergency and Pain Medicine, Ruhr University Bochum, Klinikum Herford, Herford, Germany

## Abstract

**Background:**

Sepsis-treatment is one of the major challenges in our time. Especially fungal infections play an important role in patient's morbidity and mortality. In patients with septic shock, liver function is often significantly impaired and therefore also hepatic drug metabolism is altered.

**Case Presentation:**

We report about a 56-year-old man suffering from invasive fungal infection with multiorgan failure, after complicated medical history due to symptomatic infrarenal aortic aneurysm. On the first postoperative day, a CT scan was undertaken due to massive back pain showing renal infarction on both sides. As qualitative and quantitative renal function was impaired, hemodialysis was started immediately. Subsequently, the patient developed a compartment syndrome of the left leg and underwent fasciotomy. On admission day 7, the patient presented with hematochezia leading to colonoscopy. During this procedure, an ischemic colitis was observed. As conservative treatment failed, the patient underwent Hartmann's procedure due to progredient ischemia followed by a worsening of the clinical status due to sepsis. The patient suffered from an invasive fungal infection with *Candida* spp. and *Aspergillus* spp. Systemic antifungal treatment was initiated. Although azoles are considered first-line treatment in these cases we chose the echinocandin caspofungin for its presumed lower impact on liver function compared to azoles like voriconazole or Amphothericin B. However, caspofungin is also metabolised in the liver and can cause hepatotoxic effects. Therefore we measured metabolic liver function capacity using LiMAx®and adapted the patient's dose of caspofungin to the evaluated liver function capacity to achieve an effective and liver-protective level of the active drug. After complicated medical history with 15 weeks of hospital stay, the patient was discharged in general good condition.

**Conclusions:**

To our knowledge, this is the first report that relates antimycotic drug dosing to a functional liver test. We provide a new approach for sepsis treatment considering liver function capacity to optimize dosage of hepatically metabolised drugs with potential hepatotoxic effects.

## 1. Introduction

Hepatic drug metabolism is often altered in patients with liver dysfunction. In patients with liver cirrhosis, hepatic drug clearance is significantly reduced proportionally to the grade of liver impairment. In theory, this could be due to an absolute reduction of mass of hepatocytes vs. functional inhibition of hepatocytes vs. impairment of capillary mechanisms of sinusoids vs. consequence of oxygen lack in hepatocytes [1]. The mechanisms are still poorly understood.

In patients with septic shock, liver dysfunction or liver failure with poor outcome will develop in up to 50% of the cases. The incidence of invasive fungal infections in Germany is about 6 cases/100,000 inhabitants/year. Invasive fungal infections are among the most frequently overlooked causes of death in intensive care patients [2]. Pulmonary infection due to *Aspergillus niger* has got a prevalence of 4.1% of all fungal infections [3]. There are no systematic data about bloodstream infection caused by *Aspergillus niger*. Systemic antimycotic treatment is often needed in critically ill patients suffering from invasive fungal sepsis. The intrahepatic elimination of most antifungal drugs may cause hepatotoxic effects which points to the importance of proper dosing of drugs. Echinocandins, such as caspofungin, are known to create high intracellular hepatic tissue levels. The grade of hepatotoxicity is often described by measuring the level of liver enzymes in the plasma. However, conventional laboratory tests can only provide static results.

The dynamic liver function test LiMAx® (liver maximum capacity test, Humedics GmbH, Berlin, Germany) is a tool to measure maximal liver function capacity in different clinical settings. It is an easy to handle, noninvasive bedside test that can be used on spontaneously breathing or mechanically ventilated patients. Only the hepatic, microsomally localized hemoprotein enzyme 1A2 (CYP1A2) from the cytochrome P450 group can metabolize methacetin. For testing the enzymatic liver function 2 mg/kg body weight ^13^C-labelled methacetin is given intravenously. Methacetin is subsequently metabolised into CO_2_ and paracetamol. The exhaled amount of ^13^CO_2_ is proportional to liver function capacity [4]. In correlation to time, a dynamic evaluation of liver function can be performed. An improvement of the metabolic rate may indicate early recovery of liver function prior to normalization of static laboratory parameters.

One area of application is surgery planning prior to hepatic resections to reduce severe postoperative complications [5]. In our center the LiMAx® is already well established for this application, but we propose that this test provides also a suitable tool for the diagnosis and functional assessment of liver function induced by different other causes.

Kaffarnik et al. related early diagnosis of sepsis-related hepatic dysfunction to the prognostic survival. A LiMAx® test result lower than 100 *µ*g/kg/h and respiratory dysfunction were associated with increased mortality. In the reported case, the LiMAx® value decreased significantly two days after onset of sepsis [6]. Wicha et al. recently described drug-monitoring of linezolid in critically ill patients with functional liver test using LiMAx® being superior to common markers of liver function [7]. The available data strongly suggest that liver insufficiency can be detected and quantified early and effectively using the LiMAx® test [5].

We report individualized dosing of caspofungin in a 56-year-old man with complicated medical history after emergent surgery of a symptomatic infrarenal aortic aneurysm with acute ischemia of the left leg and postsurgical bloodstream infections due to *Candida* spp. and *Aspergillus* spp. With septic liver failure. The individualized dosing of echinocandins was adapted in consideration of the current liver function capacity of the septic patient measured with a bedside test for liver failure assessment. To our knowledge, this is the first report on individualized dosing of antimycotics in relation to a functional liver test.

## 2. Case Presentation

**Figure 1:**
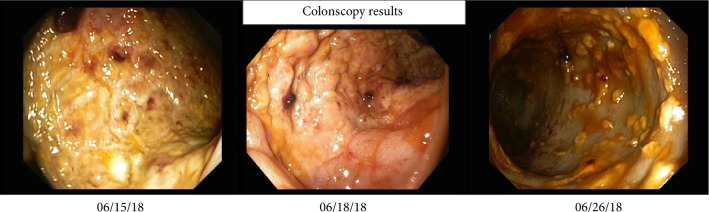
Representative colonoscopy findings in the course. Left: left-sided ischemic colitis; middle: recovery of colitis; right: severe progression of colitis with additional *Clostridium difficile* infection.

The 56-year-old patient with a body weight of 78 kilograms was assigned to hospital on 8^th^ June (hospital day, hd1) with acute pain in the left leg with preexisting intermittent claudication. Radiology diagnostics revealed an acute ischemia of the common iliac artery due to a newly diagnosed infrarenal aortic aneurysm. Therefore, the patient underwent emergency surgery with aortobifemoral prosthesis, thrombectomy, and patch of the common femoral artery and the profound femoral artery on both sides. Postoperatively, the patient was transferred to the intensive care unit with low-dose vasopressors and heparin anticoagulation with prothrombin time aimed at 50–60 sec. A prophylactic antibiotic treatment included the first generation cephalosporin cefazolin and metronidazole for three days. Respiratory weaning was uncomplicated. On the first postoperative day (hd2) the patient complained about massive back pain and pain of the legs. Another CT scan revealed renal infarction on both sides as well as an intra-aortic thrombus reaching from the left renal artery to the right renal artery above the implemented aortobifemoral prosthesis without significant impairment of the aortic lumen. Qualitative and quantitative renal function was impaired and hemodialysis was established immediately. Furthermore, the patient developed a compartment syndrome of the left lower leg and underwent fasciotomy. Presumably due to the antithrombotic treatment the patient presented with hematochezia. In a colonoscopy on 15^th^ June (hd8) a left-sided ischemic colitis was described. A colonoscopic control three days later showed recovery of the bowel function and regression of colitis. Unfortunately, by this time, diarrhea based on a *Clostridium difficile* infection was diagnosed and an antibiotic treatment with Vancomycin was started. The next colonoscopy on the 26^th^ June (hd19) revealed a severe progression of ischemia (Figure 1). The patient presented with worsening of the clinical status with septic shock and pulmonary failure, requiring mechanical ventilation. An emergency re-laparotomy with Hartmann's procedure was performed with simultaneous cholecystectomy due to a gangrene, as well as an appendectomy. The patient was in significantly reduced general condition and an abdominal compartment syndrome was suspected, so that a laparostoma with a vacuum therapy was established. Postoperatively, the patient developed septic multiorgan failure with hemodynamic instability and significant impairment of liver, kidney, and pulmonary function. Sonography of the liver excluded impairment of liver perfusion. For further diagnostics, a liver function capacity test was performed on the 2^nd^ July (hd25) measuring a LiMAx® value of 351 *µ*g/kg/h, thus excluding a significant hepatic injury. Liver enzymes were moderately elevated with ASAT 113 U/l, ALAT 84 U/l and bilirubin 1.4 mg/dL. Coagulatory function showed no abnormalities with an INR of 1.02 (Figure 3).

Beside bacterial infection from *Enterococcus faecalis*, *Klebsiella pneumonia*, *Escherichia coli*, and *Pseudomonas aeruginosa* requiring differentiated antibiotic therapy, an invasive fungal sepsis was described. *Candida glabrata* was cultured from the dialysis catheter. Therefore, an antifungal therapy with caspofungin was initiated on 5^th^ July (hd28) starting with a loading dose of 70** **mg followed by a daily dose of 50 mg (Table 1). The same day, the result of a second LiMAx® measurement was 201 *µ*g/kg/h indicating a mild liver insufficiency. Antifungal therapy was continued with 50 mg in order to maintain sufficient blood concentration.

A second septic crisis occurred on 10-11^th^ July (hd33/34) with elevation of leucocytes up to 47.7/*µ*L, CRP 141 mg/L and relapsing renal failure with the need for hemodialysis (Figure 4). Liver enzymes increased to ASAT 372 U/l and ALAT 225 U/l. This laboratory results met the criteria of potentially drug induced liver insufficiency (DILI) (>5 fold upper limit of normal) [8]. Other parameters of septic shock showed an increase of lactate (2.78 mmol/L) and interleukin 6 (746.1 pg/mL) (Figure 5). Due to clinical signs of worsening of the infection status and as a consequence of the worsening of the liver function (combination of sepsis and DILI), the dose of caspofungin was reduced to 35 mg/d on 12^th^ July (hd35) according to body weight. Subsequently sepsis was controlled, liver and kidney function recovered to previous levels. Hemodialysis treatment was stopped on 17^th^ July (hd40).

On 18^th^ July (hd41), *Aspergillus niger* was cultured from the arterial catheter. *Aspergillus* was also repeatedly found in the bronchial system. Although, voriconazole is considered as first-line therapy in this situation, but in the context of severe liver alterations, a 10-fold prolonged half-life of voriconazole has been described [9]. Therefore, we decided to continue systemic therapy with caspofungin until 11^th^ August (hd65). Only rare severe hepatic side effects are reported for caspofungin [8]. Another reason for continuing the established medication was the clinical recovery of the patient.

**Figure 2:**
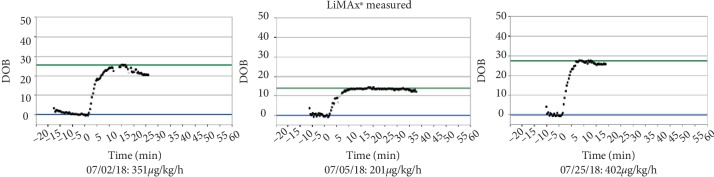
LiMAx measurements over time.

**Figure 3:**
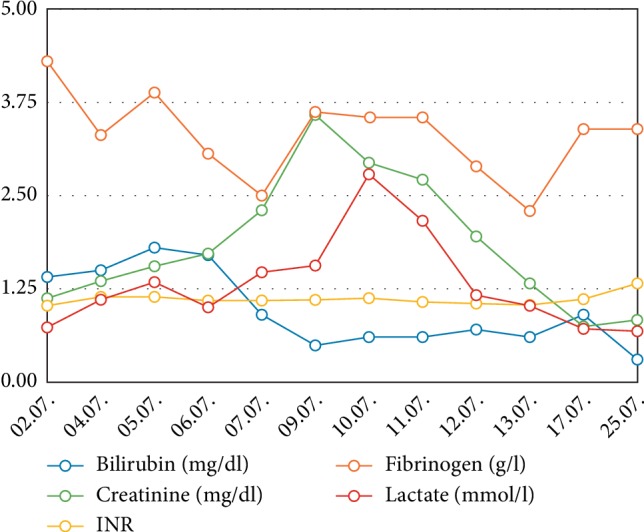
Different laboratory parameters over time. INR: International normalized ratio.

**Figure 4:**
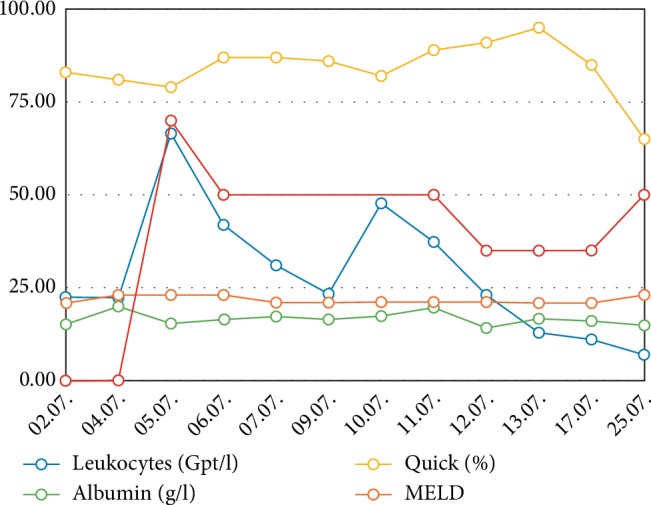
Different laboratory parameters over time. MELD: Model of endstage liver disease-score.

**Figure 5:**
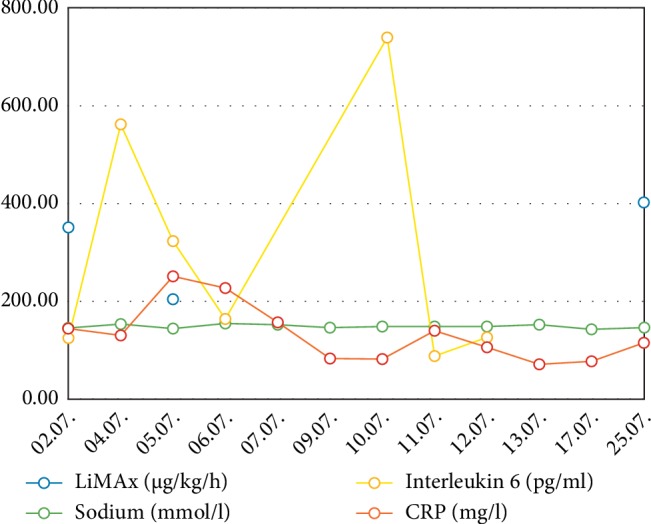
Various laboratory parameters and LiMAx measured values over time. CRP: C-reactive protein.

After recovery of liver function was confirmed on 25^th^ July (hd48) with LiMAx (401 *µ*g/kg/h), the dose of caspofungin was increased to 50 mg/d (Figure 2). After 5.5 weeks of caspofungin, the antimycotic therapy was stopped without any signs of recurrent infection. Blood cultures were negative and liver function was good. On 21^st^ August (hd75), the patient was transferred from the intensive care unit to a surgical ward.

Further minor complications included a recurrent infection with *Clostridium difficile* which was diagnosed in routine stool samples, prompting further treatment with fidaxomicin. The wounds were healed by secondary intention or using skin grafts.

Caused by the long term treatment on the intensive care unit with immobilization, the patient developed a sacral decubitus despite frequent and intensive physiotherapy. Improvement was achieved using another vacuum therapy. The patient was discharged home in good condition on 24^th^ September after 15 weeks of hospital treatment.

## 3. Discussion

Critically ill patients with septic shock develop liver failure in up to 50% of the cases whereas the liver failure is associated with poor outcome [6]. The hepatic endothelin 1-liberation mediated by proinflammatory cytokines and the sinusoidal vasoconstriction with subsequent perfusion disorder, focal ischemia, and finally liver failure are playing an important role [10]. Invasive mycotic sepsis is a special threat to critically ill patients, warranting aggressive and immediate treatment.

One of the most common antifungal agents used for treatment of infections caused by *Candida *spp. or as salvage therapy for *Aspergillus *sp. is caspofungin, an echinocandin class drug [11, 12]. These are inhibitors of 1,3 beta-glucan-synthase enzyme, which is a major component to keep the integrity of the cell wall of many fungi. Because the 1,3 beta-glucan-synthase is not formed in mammalian cells it is a “perfect target” of antifungal therapy [13]. Caspofungin is a semisynthetic derivate of pneumocandin and an essential product of the lipopeptide fermentation obtained by the fungus *Glarea lozoyensis*. Caspofungin is a potent fungicidal drug with hepatic metabolization. To reduce side effects and hepatotoxicity from caspofungin overdosing, liver function should be considered during treatment with echinocandins. The recommended loading dose of caspofungin is 70 mg/d on the first day of treatment followed by 50 mg/d. Yang et al. demonstrated that patients with liver insufficiency graded Child-Pugh-B should receive a lower dose of 35 mg/d. Maintaining a regular dose of 50 mg/d may lead to toxic blood concentrations which may result in drug-induced liver injury (DILI) [14]. Vitality, viability, and function of hepatocytes is remarkedly impaired *in vitro*, when echinocandins are overdosed [15]. The protein amount seems to matter, as 95% of caspofungin are bound to plasma proteins, especially albumin [16]. *In vitro* studies have shown that some antimycotics like fluconazole and anidulafungin have got a dose-dependent effect on albumin synthesis. High concentrations of these drugs can cause cellular necrosis [15]. Our patient with complex pathophysiological changes due to sepsis showed a significant decrease in serum albumin. At the time of dose reduction for caspofungin on 12^th^ July (hd35), the patient had a Child-Pugh score of 9 points. This was mainly triggered by the existing hypalbuminemia. The question to what extent this should lead to a dose reduction of caspofungin in order to avoid an overdose is still open [8, 14]. A potentially considered conversion to anidulafungin, another echinocandin, was not possible for us, because this drug is not listed in our hospital [16].

**Figure 6:**
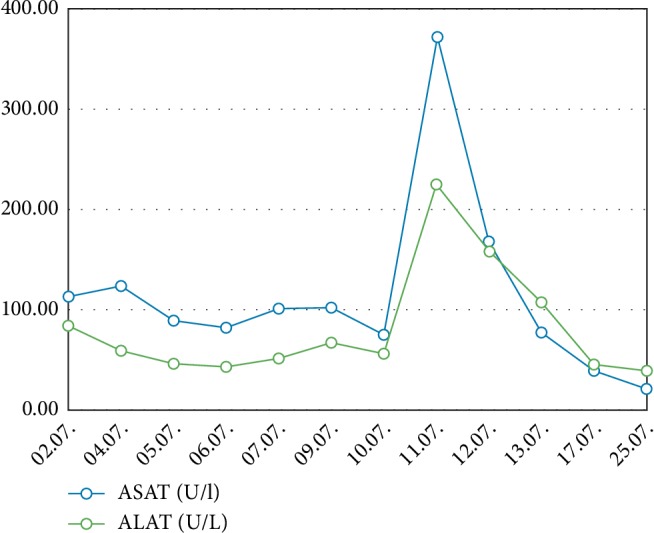
Different laboratory parameters over time. ASAT: Aspartate-Aminotransferase. ALAT: Alanine-Aminotransferase.

The summation of LiMAx®-test, the acute increase of transaminases as a sign of compromised liver integrity and the reduced serum albumin concentration led us to reduce the dose of caspofungin to 35 mg/d during treatment, followed by a rapid recovery of the transaminases in laboratory controls (Figure 6). No other significant medication was reduced or discontinued during the relevant time.

Pitfalls in measurement that may alter the LiMAx® value include continuous renal replacement therapy and liver outflow disorders (e.g. Budd Chiari), right heart insufficiency [6]. In order to exclude an influence of the continuous kidney replacement procedure on the LiMAx® measurement result, the CVVHD was paused during the measurement. Based on our former experience, these factors may be negotiated in the clinical context.

## 4. Conclusion

This case report impressively demonstrates the potential of bedside monitoring of liver function in patients suffering from septic multiorgan failure due to systemic mycosis requiring potential hepatotoxic therapy with echinocandins. LiMAx® is able to detect signs of liver dysfunction several days before the standard lab tests. This allows realtime adjustments of drug dosing considering the actual liver capacity. However, also other factors that can influence the hepatotoxicity of drugs, like the serum albumin concentration in the case of echinocandins should be taken into account.

Further studies investigating the correlation between liver function, LiMAx®-results and pharmacological impairments have to be performed to support our finding that an improved drug dosing for septic patients can be achieved considering realtime liver function capacity.

## Figures and Tables

**Table 1:** Course of laboratory values.

Value	2^nd^ July	4^th^ July	5^th^ July	6^th^ July	7^th^ July	9^th^ July	10^th^ July	11^th^ July	12^th^ July	13^th^ July	17^th^ July	25^th^ July
Caspofungin dose (mg/d)	0.00	0.00	70.00	50.00	50.00	50.00	50.00	50.00	35.00	35.00	35.00	50.00
Bilirubin (mg/dL)	1.40	1.50	1.80	1.70	0.90	0.50	0.60	0.60	0.70	0.60	0.90	0.30
Creatinine (mg/dL)	1.12	1.35	1.55	1.72	2.30	3.58	2.94	2.71	1.95	1.32	0.74	0.83
INR	1.02	1.14	1.14	1.09	1.09	1.10	1.13	1.07	1.06	1.03	1.11	1.32
Leucocytes (Gpt/l)	22.40	22.40	66.50	41.90	31.00	23.30	47.70	37.30	23.00	12.80	11.00	6.90
Albumin g/dL	15.10	19.90	15.30	16.40	17.20	16.40	17.30	19.70	14.10	16.60	16.00	14.80
Quick (%)	83.00	81.00	79.00	87.00	87.00	86.00	82.00	89.00	91.00	95.00	85.00	65.00
CVVHD (yes/no)	Yes	Yes	Yes	Yes	Yes	Yes	Yes	Yes	Yes	Yes	No	
MELD	21.00	23.00	23.00	23.00	21.00	21.00	21.00	21.00	21.00	21.00	21.00	23.00
MELD (without dialysis)	10	12	14	15	15	17	16	15	12	7	4	3
Mortality (%)	19.60	19.60	19.60	19.60	19.60	19.60	19.60	19.60	19.60	19.60	19.60	19.60
LiMAx (*µ*g/kg/h)	351.00		201.00									402.00
ASAT (U/l)	113.00	124.00	89.00	82.00	101.00	102.00	75.00	372.00	168.00	78.00	40.00	21.00
ALAT (U/l)	84.00	59.00	46.00	43.00	52.00	67.00	56.00	225.00	158.00	108.00	46.00	39.00
Sodium (mmol/L)	145.00	153.00	144.00	154.00	151.00	146.00	147.00	149.00	149.00	152.00	143.00	146.00
Interleukin 6 (pg/mL)	124.70	561.70	323.00	165.20			746.10	87.60	126.10			
CRP (mg/L)	144.30	130.10	250.80	226.90	157.80	84.10	80.20	141.00	105.70	70.80	77.40	114.90
Fibrinogen (g/L)	4.30	3.31	3.88	3.06	2.49	3.61	3.54	3.54	2.89	2.29	3.39	3.39
Lactate (mmol/L)	0.73	1.11	1.34	1.01	1.47	1.56	2.78	2.16	1.17	1.02	0.71	0.68
